# Gestational exposure to the great Chinese famine: early life undernutrition impact on prostatic hyperplasia in adulthood

**DOI:** 10.3389/fnut.2024.1391974

**Published:** 2024-06-20

**Authors:** Siyao Wang, Yong Zhang, Xiaoya Qi, Xiaoyang Xu

**Affiliations:** ^1^Department of Health Medicine Center, The Second Hospital Affiliated to Chongqing Medical University, Chongqing, China; ^2^School of Public Health, Chongqing Medical University, Chongqing, China

**Keywords:** benign prostatic hyperplasia, famine exposure, Chinese men, nutrition, hyperplasia

## Abstract

**Introduction:**

Benign prostatic hyperplasia (BPH) is a frequent illness in aged men that impacts their quality of life; early childhood exposure to famines may have long-term effects on the chance of developing BPH. The aim of this study is to investigate the relationship between early-life famine exposure and benign prostatic hyperplasia (BPH) risk in Chinese men born during 1959–1961.

**Methods:**

We used medical records from a large, comprehensive hospital to screen people born in China during the years of famine (1959–1961). Birthplaces were identified as indicators of famine exposure status. In the time window between 2017 and 2022, people born during the famine years who had prostatic ultrasonic examinations were selected, and their medical records were retrieved from the database. Univariate and multivariate logistic regression analyses investigated the relationship between famine exposure and BPH risk.

**Results:**

A total of 3,009 subjects were included in this study. Patients with heavy famine exposure had older age, shorter height, lighter weight, lower cholesterol, lower uric acid (UA), lower aspartate aminotransferase (ALT), and a higher incidence of BPH than those with light famine exposure (all *p* < 0.05). Univariate logistic regression showed that BPH was positively related to famine exposure, age, height, weight, and body mass index (BMI) but negatively related to UA (all *p* < 0.05). Multivariate logistic regression showed that age and famine exposure were still independent risk factors (*p* < 0.05), while UA was an independent protective factor for BPH (*p* < 0.05). Heavy famine exposure increased the risk of BPH (adjusted OR = 1.214, 95% CI = 1.05–1.467, *p* = 0.045).

**Conclusions and recommendation:**

Famine and malnutrition exposure during early life may be independent risk factors for BPH in Chinese adults. This relationship provides additional evidence to support the fetal origins of adult diseases and offers clues for the pathological mechanisms of BPH.

## Introduction

Benign prostatic hyperplasia (BPH) is an age-related medical condition in which the prostate gland enlarges (1). BPH usually causes bladder tract obstruction, produces lower urinary tract symptoms, decreases the quality of life, and increases health costs. BPH is exceptionally prevalent in older adults. As people live longer than before, BPH’s economic and health burden will keep rising in the coming years. Some studies suggested that of the estimated 94 million cases of BPH in 2019 globally, up to 50% of men in their 60s and 80% of men in their 90s were affected by BPH (2).

The etiology and pathogenesis of BPH are not fully understood, but it is thought to be related to sex hormonal level changes and unbalance as men aging (3). Some risk factors for BPH include family history and certain medical conditions such as obesity, metabolic syndromes, hypertension, and diabetes (4). Lifestyle changes, such as diet modification, more physical activity (5), and some nutrient supplements, can reduce the risk of BPH (6). Because of the close relationship between BPH and metabolic conditions, some researchers have named BPH a new type of metabolic disease (7, 8).

From the metabolic diseases point, many studies have shown that early-life famine exposure, including *in utero* or after birth, increases the prevalence or risk of different metabolic diseases in adults (9). For example, Wang J et al. found that adults who had gestational exposure to Chinese famine were 1.5 times more likely to have diabetes at their 60s (10). Another study in rural Bangladesh showed that young adults with gestational exposure to famine have higher risk of hyperglycemic than those unexposed (11). In addition, animal studies also found that maternal protein malnutrition delayed the prostate’s (12) and epididymal’s (13) development, growth, and maturation, which suggests that a link between early life nutrition status and the development of BPH may also exist (14). We, therefore, hypothesized that malnutrition exposure in the fetal stage can also increase the risk of BPH in midlife adults.

Famines in human history were usually used as a natural experiment to study the effects of early life malnutrition on health and disease in adults, also called the developmental origins of health and disease hypothesis (DOHaD) (15). The Chinese famine, also called the Great Famine, was a national disaster that happened in 1959–1961 and caused millions of people to die because of severe food shortages (16). Due to its severity, pervasiveness, and long duration, Chinese famine has been extensively studied in many aspects, such as the effects of famine on overweight, obesity, type 2 diabetes, hyperglycemia, hypertension, metabolic syndrome, and schizophrenia in adults (17). However, to our knowledge, no such study has been conducted on BPH. Therefore, this study aims to investigate the association between fetal famine exposure and the risk of BPH in adulthood by using a population exposed to the Chinese famine.

## Methods

### Study design

This study was a cross-sectional study. Routine health check-up records from the second hospital affiliated with Chongqing Medical University in the years 2017–2019 were used. This study was approved by Chongqing Medical University. The ethic approval paper’s number is no. 2020-252.

The de-identification data from medical records used in this study.

### Participants and data retrieval

The subjects were relatively healthy when they underwent their routine health examination. According to the literature, the Great Chinese Famine peaked in 1959–1961 (18). In the time window between 2017 and 2022, people born during the famine years who had prostatic ultrasonic examinations were selected, and their medical records were retrieved from the database. Only the most recent examination was valid for this study when a person had multiple prostatic examinations with consistent or inconsistent results. After that, the unique ID of the subjects will be used to link the data from physical and biological examinations. Physical examinations and serum biological tests were conducted and recorded following standard procedures by qualified personnel in the hospital.

### Fetal famine exposure intensity definitions

Due to the rigid household registration system in China (19), we can extract the birthplace code from the 18-digit ID numbers for each person at the county level. Subjects were categorized by birthplace into two groups: one for those born in Chongqing city and the other for those born in rural areas around Chongqing city. Because of the food rationing system before China’s economic reform in 1978, city residents were given the legal right to acquire a certain amount of food, which caused an unequal distribution of food between urban and rural areas (20). Therefore, those born in rural areas suffered the most during the famine. On the contrary, those who were born in the city of Chongqing suffered less compared to their rural counterparts during the famine. Accordingly, subjects were defined as having light fetal famine exposure when born in Chongqing City or heavy fetal famine exposure when born in rural and remote areas around Chongqing City.

### Statistical analysis

In the statistics description, continuous variables such as age, height, weight, blood pressure, were expressed as mean ± standard deviation; category variable, i.e., the year of birth, were expressed as N and percentage. The comparisons among different famine exposure groups were conducted by the t-test, ANOVA (Analysis of Variance) for continuous variables, or Chi-squared test for category variable. In exploring of risk factors (independent variables) of BPH (dependent variable), the univariable logistic regression analysis was used. To evaluate the relationship between gestational famine exposure and BPH, multivariable logistic regression analysis was used to adjust the influences of confounders. In the sensitive analysis, samples born out the famine periods, i.e., born before or after the famine, were used to test the possible influence of the birth places on the risk of BPH by using logistic regression analysis. The statistical significance level was considered *p* < 0.05. All data processing and analysis used statistical software, SPSS 24.0.

## Results

### Characteristics of subjects

During 2017–2022, 4,447 records of prostatic examination were identified by limiting the birth year to famine, i.e., between 1959 and 1961. After deduplicating the ID number and excluding those with missing data, 3,009 subjects were included in this study. Among them, 655 (21.8%) were born in rural areas and classified as having heavy fetal famine exposure. On the contrary, 2,354 (78.2%) were born in Chongqing City and classified as having light famine exposure because their mothers suffered relatively less than their rural counterparts.

These subjects born during famine reached their midlife age of around 60 when taking a medical examination in this study. The characteristics measured during 2017–2022 are presented in Table 1. Data showed that those with heavy famine exposure during the fetal stage were shorter (*p* = 0.001) and lighter (*p* = 0.144), with a more significant BMI (*p* = 1.151). In addition, subjects suffering heavy fetal famine exposure were low in serum cholesterol (*p* = 0.015) and uric acids but high in liver enzymes such as AST and ALT (*p* = 0.089).

**Table 1:** The characteristics of subjects by fetal famine exposure intensity.

	Heavy fetal famine exposure (born in rural area) (*n* = 655)	Light fetal famine exposure (born in Chongqing city) (*n* = 2,354)	*p* value
Birth year			0.190
1959	223 (34.0)	739 (31.4)	
1960	209 (31.9)	839 (35.6)	
1961	223 (34.0)	778 (33.1)	
Age (year)	59.32 ± 1.73	59.02 ± 1.68	>0.001
Height (cm)	164.72 ± 6.12	166.1 ± 36.18	>0.001
Weight (Kg)	67.40 ± 9.57	68.01 ± 9.2	0.144
BMI (kg/m2)	24.79 ± 2.86	24.61 ± 2.81	0.151
SBP (mmHg)	130.77 ± 17.47	130.58 ± 17.43	0.805
DBP (mmHg)	80.11 ± 11.61	79.21 ± 11.09	0.074
TC (mmol/L)	5.02 ± 0.93	5.13 ± 0.99	0.015
LDL (mmol/L)	2.72 ± 0.72	2.77 ± 0.76	0.192
UA (μmol/L)	366.96 ± 77.96	375.91 ± 83.19	0.015
AST (IU/L)	24.86 ± 18.41	23.15 ± 12.72	0.007
ALT (IU/L)	27.64 ± 51.48	25.48 ± 17.08	0.089
BPH (yes)	291 (44.4%)	923 (39.2%)	0.016

BMI, body mass index; SBP, systolic blood pressure; DBP, diastolic blood pressure; TC, total serum cholesterol; LDL, low-density lipoprotein; UA, uric acid; ALT, alanine aminotransferase; AST, aspartate aminotransferase; BPH, benign prostatic hyperplasia. Mean (SD) for Continuous data; where categorical, data are *n* (%).

Furthermore, the overall prevalence of BPH was 40.3%, as 44.4% of subjects with heavy fetal famine exposure were detected to have BPH, significantly higher than those with light fetal famine exposure (39.2%, *p* = 0.016) (see Table 1).

### Univariate analysis of the factors of BPH

In the univariate analysis, age is the most substantial factor positively related to BPH. Besides, the intensity of fetal famine exposure, height, weight, and BMI were positively associated with the risk of BPH (all *p* < 0.05), and Blood pressures were unrelated to BPH. Interestingly, TC, LDL, and UA were all negatively associated with BPH, but only UA reached a significant level (*p* = 0.021); liver enzymes, i.e., ALT and AST, were not associated with BPH (see Table 2).

**Table 2:** The univariate logistic regression analysis of risk factors for BPH.

	*B*	se	*p*	OR	95%CI of OR
**Fetal famine exposure intensity**					
Light (reference)				1	
Heavy	0.215	0.089	0.016	1.239	1.041–1.476
Age (year)	0.135	0.022	<0.001	1.144	1.096–1.195
Height (cm)	0.02	0.006	0.001	1.021	1.008–1.033
Weight (Kg)	0.015	0.004	<0.001	1.015	1.007–1.023
BMI (kg/m2)	0.028	0.013	0.039	1.028	1.001–1.056
SBP (mmHg)	0.001	0.002	0.729	1.001	0.997–1.005
DBP (mmHg)	0.003	0.003	0.398	1.003	0.996–1.009
TC (mmol/L)	−0.022	0.038	0.566	0.978	0.908–1.054
LDL (mmol/L)	−0.031	0.051	0.535	0.969	0.878–1.07
UA (μmol/L)	−0.001	>0.001	0.021	0.999	0.998–1.000
AST (IU/L)	−0.002	0.003	0.452	0.998	0.992–1.003
ALT (IU/L)	<0.001	0.001	0.777	1.000	0.998–1.003

BMI, body mass index; SBP, systolic blood pressure; DBP, diastolic blood pressure; TC, total serum cholesterol; LDL, low-density lipoprotein; UA, uric acid; ALT, alanine aminotransferase; AST, aspartate aminotransferase.

### Multivariable analysis of fetal famine exposure and BPH

When adjusting for all potential factors in the logistic analysis, the intensity of fetal famine exposure was still significantly related to the risk of BPH. The 95% CI of the OR is 1.005–1.467 (*p* = 0.045). Note that metabolic variables have no relationships with BPH except uric acid, which is significantly negatively related to BPH (see Table 3).

**Table 3:** The fetal famine exposure and the of risk for BPH.

	*B*	se	*p*	OR*	95% CI
**Fetal famine exposure**					
Light (reference)				1	
Heavy	0.194	0.097	0.045	1.214	1.005–1.467

*Logistical analysis adjusted by BMI body mass index; SBP, systolic blood pressure; DBP, diastolic blood pressure; TC, total serum cholesterol; LDL, low-density lipoprotein; UA, uric acid; ALT, alanine aminotransferase; AST, aspartate aminotransferase.

### Sensitive analysis

**Figure 1:**
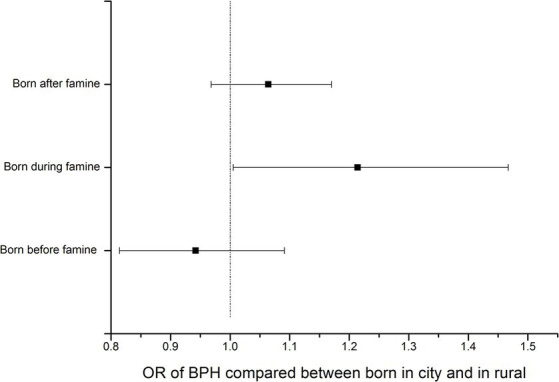
The ORs and 95% CI of BPH in population born before, during and after famine.

Birthplace, used as a surrogate for famine exposure intensity in this study, may also affect BPH due to differences beyond famine exposure. We, therefore, conducted a sensitivity analysis to test the effects of birthplace on BPH in the non-exposure population from the same database. Data showed that the birthplace did not affect BPH in populations born closely before or after the famine (*p* > 0.05) (Table 4; Figure 1).

**Table 4:** The sensitive analysis of birthplace on BPH in those born before or after famine.

	*B*	se	*p*	OR	95% CI
**Born before famine (1956–1958)**					
Born in Chongqing city (Reference *n* = 2,920)				1	
born in Chongqing rural area (*n* = 956)	−0.060	0.075	0.425	0.942	0.814–1.091
**Born after famine (1962–1964)**					
Born in Chongqing city (Reference, *n* = 6,047)				1	
Born in Chongqing rural area (*n* = 2,667)	0.062	0.048	0.198	1.064	0.968–1.170

## Discussion

BPH is a disease of aging and causes deterioration of quality of life through complex symptoms in older people, such as lower urinary tract symptoms, nocturia, and bladder outlet obstruction (21), which causes a considerable burden on health and economics (22). BPH’s initiating factor(s) have yet to reach a consensus. Androgens, oestrogens, an increased estrogen/androgen ratio, and decreased levels of Erα receptor have been recognized as possible causes of BPH (23), as well as hyperinsulinemia and diabetes as risk factors (24). The overall prevalence of BPH among men aged 40 years and older was estimated to be 36.6% (25).

In this study, all the subjects we included were born in 1959–1961, the peak year of the Chinese famine. They were grouped into heavy fetal famine exposure and light fetal famine exposure according to their birthplace (rural vs. city). The overall prevalence of BPH was 40.3%, as it was 44.4 and 39.2% for heavy and light famine exposure groups, respectively, which was close to the study by Wang et al. (25). After adjusting all confounders available in this study, we found that heavy famine exposure increased the risk of BPH moderately and significantly (adjusted OR = 1.214, 95% CI = 1.005–1.467) when comparing to light famine exposure. This study is the first study in humans that confirms our hypothesis that early-life malnutrition exposure in fetuses can increase the risk of BPH. These results implied that environmental insult in the early stage of life could also impact prostate disease, which tested the DOHaD (developmental origins of health and disease) hypothesis in BPH disease.

Famine caused by the severe shortage of food can lead to malnutrition. In the fetal or early childhood stage, it can directly cause growth retardation. Beyond that, early life malnutrition can also reprogram the development, metabolism and endocrine by epigenetic modification (26) to adapt to unfavorable environments, which may enhance susceptibility to diseases in a standard or affluent environment. Therefore, birth weight, as an indicator of fetus malnutrition, was found to be related to adults’ metabolic diseases, such as overweight/obesity, hypertension, diabetes, and dyslipidemia, hyperuricemia, non-alcoholic fatty liver, cardiovascular diseases (17). It is worth noting that in this study, metabolic indexes such as BMI, blood pressure and, serum lipids LDL, liver enzymes ALT had no relationship with famine exposure or the risk of BPH, which may imply that BPH is not all the secondary outcome of metabolic disorder regarding famine exposure. In other words, famine exposure may influence metabolic diseases and BPH independently by some different pathways. The embryonic stem cell reawaking hypothesis was an alternative explanation for prostate enlargement (23, 27). However, these mechanisms need to be elucidated by studies.

Interestingly, we also found that uric acid, another metabolic index, was negatively and significantly related to the famine exposure level and the risk of BPH. This finding was contrary to a study that showed gout patients have a high incidence of BPH, especially in young gout patients but not those beyond 60 (28). However, this result was consistent with another study from Korea showing that higher uric acid was associated with a decreased risk of lower urinary tract symptoms (29). The underlying biological mechanism needs further study to clarify.

In studies related to Chinese famine, age-balanced controls and robust famine intensity measures are both critical issues that made the results inconclusive, as found in systematic reviews (13, 17). The way to eliminate the impact of age is to find an indicator that can distinguish the famine status from inside of a population born in the same years. That is not easy because many clues have disappeared or become vague when people get old. In a study, the authors used the subjects’ parents’ party membership as the famine exposure severity indicator to investigate the impact of fetal famine exposure on perceived health in adults (18). In this study, we creatively chose the birthplace for the intensity of famine exposure. In the centrally planned economics era before the 1990s, China had a rigid household registration system (20). From the ID number, we can trace back the birthplace of a citizen. At the same time, there was a food rational system under which city residents had legal protection in acquiring the amount of food (20). Therefore, birthplace can surrogate famine exposure intensity in famine year. Our sensitivity analysis of birthplace showed that the birthplace did not relate to the prevalence of BPH in adults born before or after the famine, which implied that the birthplace as a socioeconomic factor could be a valid surrogate of the severity of famine exposure. Therefore, this study provides new and solid evidence to support the DOHoD hypothesis by testing a new type of disease and clues to explore the potential etiology and pathological mechanism of BPH.

Regarding the strengths, this is the first time to confirm the early life exposure to malnutrition on the risk of BPH in humans, which provided direct evidence in humans. In this study, we adopted a reliable indicator to distinguish the famine exposure status and, by doing this, avoided the age bias that commonly existed in other Chinese famine studies. Third, the study was based on medical records, which are more reliable than questionnaire data. However, there are some limitations of this study: First, Subjects come from one hospital, which may cause selection bias, and the results may not be generalized to other populations; a multicentral study is recommended in future. Second, not all potential confounders were available; their influences on BPH cannot be entirely excluded from the results of this study. Third, this study is limited to Chinese people; the results may not apply to other ethnic people.

## Conclusion

In conclusion, the results of this study showed that famine and malnutrition exposure during early life may be independent risk factors for BPH in Chinese adults. This relationship provides additional evidence to support the fetal origins of adult diseases and offers clues for the pathological mechanisms of BPH.

## Data availability statement

The raw data supporting the conclusions of this article will be made available by the authors, without undue reservation.

## Ethics statement

The studies involving humans were approved by Chongqing Medical University. The ethic approval paper’s number is no. 2020-252. The studies were conducted in accordance with the local legislation and institutional requirements. Written informed consent for participation was not required from the participants or the participants’ legal guardians/next of kin because de-identification data from medical records used in this study.

## Author contributions

SW: Writing – review & editing, Writing – original draft. YZ: Writing – review & editing, Writing – original draft, Formal analysis, Data curation, Conceptualization. XQ: Writing – review & editing, Writing – original draft. XX: Writing – review & editing, Writing – original draft, Formal analysis, Data curation, Conceptualization.
